# A Naturalistic Observation of Spontaneous Touches to the Body and Environment in the First 2 Months of Life

**DOI:** 10.3389/fpsyg.2018.02613

**Published:** 2018-12-18

**Authors:** Abigail DiMercurio, John P. Connell, Matthew Clark, Daniela Corbetta

**Affiliations:** Department of Psychology, The University of Tennessee, Knoxville, Knoxville, TN, United States

**Keywords:** touch, self-touch, infancy, embodiment, sensorimotor experience, emerging self

## Abstract

Self-generated touches to the body or supporting surface are considered important contributors to the emergence of an early sense of the body and self in infancy. Both are critical for the formation of later goal-directed actions. Very few studies have examined in detail the development of these early spontaneous touches during the first months of life. In this study, we followed weekly four infants in two naturalistic 5-min sessions (baseline and toys-in-view) as they laid alert in supine from the age of 3 weeks until they acquired head control. We found that throughout the 2 months of observation, infants engaged in a high rate of touch and spent about 50% of the time moving their hands from one touch location to the next. On most sessions, they produced up to 200 body/surface contacts and touched as many as 18 different areas (mainly upper body and floor) both hands combined. When we did not consider the specific areas touched, the rates of touches were higher to the body than to the floor, but the duration of contacts and the most touched areas were higher for the supporting surface than for the body. Until the age of 9 weeks, we found no consistent differences in the rate of touch between head and trunk. Infants also did not display significant differences in their rate of touch between right and left hand or between conditions. However, we discovered that in the earlier weeks, infants engaged more often in what we called “complex touches.” Complex touches were touches performed across several body/floor areas in one continuous bout while the hand maintained contact with the body or floor. Single touches, in contrast, corresponded to one touch to one single body or floor area at a time. We suggest that infants are active explorers of their own body and peripersonal space from day 1 and that these early self-generated and deeply embodied sensorimotor experiences form the critical foundation from which future behaviors develop.

## Introduction

Developing a sense of our body is an essential prerequisite for our interactions with the world. Sensing our body entails knowing where our limbs are in space and time, being aware of how fast or how far our limbs can move, or even knowing how much space our body occupies in our proximal environment. Indeed, knowing the limits and extent of our peripersonal space is fundamental for navigating our social and physical world, and for situating and orienting ourselves in our everyday activities. Little is known about how such sense of the body develops in early infancy. Many pioneers in developmental psychology like Piaget (1936/1952) and Wallon (1941) initially assumed that newborns lived in complete adualism during the first months of life, meaning that newborns were assumed unable to differentiate their own body from their surrounding world. According to these pioneers, the development of a sense of the body and awareness of a person’s self would take months and even years to build. Nowadays, however, many researchers are acknowledging that an emerging sense of the body – a precursor of the sense of self – already begins to form in the womb (Reissland and Austen, 2018), can be observed at birth (Meltzoff and Moore, 1977; Filippetti et al., 2015) and is certainly present and detectable at 2 months of age (Bahrick, 1995; Rochat, 1995, 1998). Yet, systematic assessments of how such emerging sense of the body develops from birth to 2 months of life are generally lacking.

The goal of this study is to begin to examine infants’ spontaneous contacts with their own body and supporting surface from 3 weeks to 2 months of life. This early period of development is critical because this is a time during which infants perform self-generated activities almost entirely within their peripersonal space and because these initial free form behaviors and contacts may be important contributors to the formation of body map representations and later goal-directed behaviors (Corbetta et al., 2014; Marshall and Meltzoff, 2015; Thomas et al., 2015). Indeed, prior to the onset of reaching, which occurs around 3–5 months of age, infants are greatly restricted in what they can do. As a result, they are often perceived as passive and dependent organisms that are limited to the sensory and physical experiences that happen in their immediate vicinity. Despite these limitations, development and learning is at work from day 1, and discovering the body via self-generated movements of the arms and legs is probably one of the earliest behaviors to which infants attend around the clock. Infants and even fetuses experience touch through spontaneous limb activity resulting in contact with their own bodies or their immediate environments (Thomas et al., 2015; Fagard et al., 2018). In fact, many early experiences center around touch, as touch is often considered one of the first senses to develop (Field, 2014; Reissland and Austen, 2018).

In this paper, we document the spontaneous touch activities of 4 infants that we followed longitudinally, every week, until they developed head control (between 9 and 13 weeks of age). Our goal was to provide a detailed description of the early development of infants’ touches to their own body and supporting surface in order to gain a better understanding of how these early body/environment-oriented sensorimotor experiences might contribute to the development of an initial sense of the body, a necessary precursor to the emergence of reaching and subsequent goal-directed behaviors.

### The Emerging Sense of the Body

The ability to sense our body is intimately linked to self-perception. Self-perception can be proprioceptive as we move our limbs and head in space and time, and it can be haptic as we touch a surface or our own body. These deeply embodied sensorimotor experiences can occur in conjunction with other perceived information, such as turning the head toward a sound or looking at and/or tracking a moving object. Newborn infants spontaneously perform these activities from birth, on a day-to-day, second-by-second basis. At each moment, they are recipients of proprioceptive and haptic feedback that informs them about their posture, any changes in limb position, about contact with themselves or other surfaces, thereby allowing them to discover not only their limbs and their range of motion, but also the limits of their peripersonal space. These self-generated movements, as newborns move their limbs freely, clearly provide a foundation for exploratory behavior from which body representation and a basic, implicit form of self-knowledge build (Hoffmann, 2017). Particularly, touches to the body may provide redundant information about the limb posture in space, the part of the limb making contact with the body, and the body area being touched (Rochat and Hespos, 1997). These particular touches may differ from those where the limbs only touch the supporting surface on which the body lays. Indeed, these later touches may provide more specific information about limb extensions and their range of activity within the infant’s peripersonal space.

Studies that have examined spontaneous and exploratory motor activities in early development have mainly focused on the prenatal, neonatal, and later months of the 1st year of life. Studies on prenatal development have shown that very early on, fetuses already direct their arms toward their body and face (Piontelli, 1987). Self-touches on the body and face increase in the last months of gestation and are accompanied by increasing gross body movement activity and increasingly complex and frequent limb movements (Andonotopo et al., 2004). Some other studies even provided evidence that an emerging sense of the body may already exist in the womb (Zoia et al., 2007; Reissland et al., 2014). These later studies reported that fetuses generate ample spontaneous limb movements, however, when limbs are approaching the mouth, it was observed that the speed of the limb movements decreased, compared to other limb movements directed toward other parts of the face, like the eyes (Zoia et al., 2007). Fetuses were also found to open their mouth in anticipation of their hand making contact with the mouth, suggesting that they may have had a basic body representation of where the hand was being directed (Myowa-Yamakoshi and Takeshita, 2006). These authors often argue for an early form of action planning based on spatial awareness and body knowledge (see also Reissland and Austen, 2018). However, the mouth may be a unique case and caution may be required in interpreting these prenatal movements as “goal-directed” or “prospective” (see Delafield-Butt et al., 2018). Indeed, other evidence has shown that infants at 3 months of postnatal age do not succeed in reaching toward their arms, hands, legs, or feet when prompted by visuo-tactile stimuli (Somogyi et al., 2018). In sum, observations of prenatal behavior reveal that self-touch is already very active in the womb and that these body oriented spontaneous behaviors, providing both proprioceptive and haptic information within the same time frame, may already begin to contribute to the emergence of an early sense of the body (Bahrick, 1995).

At birth, a slight regression in motor activity can occur as neonates adjust to the new ambient gravitational field, compared to when motor activity was performed in the amniotic fluid (Fagard et al., 2018). This transition typically translates into a slight decrease in nearly all hand to body self-touch activities, aside from hand-to-mouth movements which increase in postnatal life (Kurjak et al., 2004). Prematurely born infants also tend to move their hands to their head, the only part of their body not covered in clothing (Durier et al., 2015). The authors suggested that this postnatal increase in self-touch activity to the head could be related to self-soothing responses which again could be interpreted as evidence of an emerging sense of the self. Neonates have also been shown to imitate certain gestures (e.g., tongue protrusion, hand opening/closure) when modeled by an adult (Meltzoff and Moore, 1977; Vinter, 1986), and very recently, researchers have identified movements of the arms in few-hour-old full-term neonates that presented kinematic profiles consistent with those of movements performed prospectively, that is, similar to goal-directed patterns (Delafield-Butt et al., 2018). However, in this study, they also found that 25% of the responses were not meeting the authors’ criteria for movement “prospectiveness,” which caused the researchers to caution about the functionality of these early motor responses. Interestingly, in this same study, the researchers found that prospective arm activities were much disrupted in infants born preterm.

Clearly, more studies are needed to understand how prenatal motor activity relates to post-natal motor activity. Furthermore, to fully understand the functional context of self-touch activity and possible movement prospectiveness, these early behaviors should be studied from a dynamic systems perspective, that is within the realm of multiple developing systems such as hunger, comfort, motor ability, environmental stimulation, caregiver presence, and more, to assess variations in behavior and gain deeper insights into the meaning of these early movement activities.

When we turn to studies performed with older infants, reports evidencing a form of awareness of the body and limb movements become more frequent, especially in studies performed with infants aged 2 months and older. Investigations using contingent reinforcement in the mobile kicking paradigm have revealed that infants as young as 10 months old can modify their rate of kicking to increase the motion of a mobile that is tied to one of their legs (Rovee and Rovee, 1969). This change in kicking response indicates that they are capable of recognizing the contingency between their leg movements and the action of the mobile. Three-month-old infants were also shown to increasingly choose harder-to-produce simultaneous kicking of both legs to receive the contingent reinforcement (Thelen, 1994). In another variation of this leg kicking paradigm, Angulo-Kinzler and colleagues demonstrated that 3-month-old infants could even discover how to adopt specific leg postures or specific hip and knee angles to increase the mobile activity (Angulo-Kinzler, 2001; Angulo-Kinzler et al., 2002). Similar contingency discovery was additionally observed in 2-month-old infants when the mobile was attached to their arms, instead of their legs (Watanabe and Taga, 2006).

Other studies have shown that infants can detect incongruences between leg movements they produce and the filmed images of their own leg movements (Rochat and Morgan, 1995; Rochat, 1998). In those studies, when infants were shown inverted recordings of their actual leg movements (e.g., the right leg was moving on the TV monitor while infants were, in fact, moving their left leg), infants as young as 3 months old looked longer at the incongruent video than the congruent one. Along the same vein, studies on tactile stimulation (Bremner et al., 2008; Begum Ali et al., 2015) have revealed that between the ages of 4 and 6 months, infants are more likely to rely on haptic stimulation to select a limb when the limbs are crossed, compared to older infants who are slower and prone to more errors in limb selection. Presumably, the older infants are confused by the fact that haptic sense and the spatial representation of the source of stimulation do not match when the limbs are crossed. This is a puzzling finding, especially knowing that the body maps for the hands in the somatosensory area of the brain of 60-day and 7-month-old infants appear to be lateralized (Marshall and Meltzoff, 2015; Saby et al., 2015; Meltzoff et al., 2018a,b).

These studies as a whole clearly suggest that infants have developed a basic sense of their body by the age of 2 or 3 months. They can select and activate the limb that creates an interesting event or that corresponds to a lateralized haptic source of stimulation, they demonstrate a sense of agency, and attend more to the events that do not match the outcome of their actions. In a more recent study, however, using a different paradigm, body self-knowledge around that same age range appeared to be lacking. This study used tactile stimuli in the form of “pancake buzzers” that were placed on specific limbs or body areas of infants with widely varied reaching experiences (Somogyi et al., 2018). The buzzers produced small vibrations on the infants’ skin and were also clearly visible to the infant depending on the body placement. In 3-month-old infants, the buzzer generated increased, generalized body activity that was non-specific to the location of the buzzer. Based on the studies reviewed above, one could assume that by 3 months of age, infants have acquired sufficient self-touch and limb movement experience to differentiate limb activity. Yet, from those findings, it remains unclear why at that age undifferentiated activity occurred.

From this brief review, it appears that self-touch activity takes place well before birth and intensifies as the fetus reaches the last gestational period. Observations of the limb movements of fetuses and neonates suggest that they may have begun to acquire an initial sense of their body. This sense of the body is becoming more evidenced from the age of 2 months and beyond, when infants demonstrate that they are capable of producing more targeted movement reactions in responses to specific stimulations or contexts involving specific parts of their body. However, studies examining in detail the limb movement activity of young babies in the first 2 months of their life are lacking. This is an important omission, as this period marks a time during which infants are adapting to their new airborne environment (Fagard et al., 2018). Newborn vision is also very poor, limiting their apprehension of the more distant extra-personal space. Therefore, much of their sensory and motor experience is centered on their body and the surface surrounding their body’s limits. These deeply embodied first 2 months of life not only provide continuity between the early body sensations experienced in the womb and the more targeted responses of older infants, but also contribute greatly to the infants’ journeys of discovering what they can do with their body and how they can situate themselves in the environment.

### This Study

The present study aims to examine the naturalistic progression of infants’ spontaneous touches to their body and supporting surface from the time they are 3 weeks old until they have acquired head control (between 9 and 13 weeks of age). We observed infants weekly, while in supine, over two 5-min sessions varying only by the presence or absence of objects in their visual field. During that age span, most infants in western cultures spend a large amount of time in the supine position while in their cribs or play-pens. Therefore, studying self-touch in this context allowed us to examine the behaviors that infants would most likely exhibit and experience during that early age range.

This is a descriptive study that is part of a larger longitudinal study where we followed a few infants at close weekly intervals until they were able to reach for objects. In this report, we focused specifically on the first 2 months of life preceding the emergence of head control. The emergence of head control marks an important transition in the perceptual and motor development of the infants and provides a critical foundation to the formation of eye, head, and trunk control that is needed for object reaching (Bertenthal and von Hofsten, 1998). In our study design, when infants demonstrated head control, we no longer observed them while in supine; we moved them into a different paradigm, where they were supported on a seat, in order to capture reaching onset. In the present report, we concentrate on two supine conditions: a baseline condition, and a toys-in-view condition, where colorful toys were placed on the side of the infant preferred head turn. For each session, we asked how many touches infants performed during each 5-min observation, which part of their peripersonal space they touched most – their body or the supporting surface – or if they spent more time moving their arms in the air than touching their body. We documented which and how many different areas of their body they touched in one session, for how long, and if there were differences between right and left arms.

To our knowledge, only one study examined self-touch behavior in infants from birth to 24 weeks of age (Thomas et al., 2015). These researchers observed self-touch over 21 s (on average) of video recordings and limited their behavioral analyses to the first 10 self-touch observed. They also divided the body into 3 major areas: the head, torso, and legs, with detailed analyses of the hand posture during self-touch (i.e., palmar or dorsum contacts). The authors found that infants followed a cephalocaudal progression with more touches to the head and torso at first, followed by more touches to the legs by 12 weeks of age (Thomas et al., 2015).

The present study complements this prior work by providing detailed behavioral observations of fewer infants, but over segments of 5-min-weekly observations. We coded every touch performed in relation to a more detailed map of the body using a transition network to track where the hands moved from place to place on the body, including contacts with the supporting surface. We also controlled the position of the infants by using the posture that seemed the most ecologically valid for the age range studied and the one in which infants naturally explore and experience their body the most. Finally, we manipulated the environment of the infants by introducing colorful objects in the infants’ view in one condition. The introduction of colorful objects in the infant view was aimed to assess whether perceiving objects would affect the patterns of touches to the body and surface. In particular, we thought that as infants would develop visual attention, especially in the later weeks when they approached 2 months of age, we could eventually observe a slowing down of movement of the arms since at that time infants could be expected to stare more at the objects (Colombo, 2001).

## Materials and Methods

### Participants

Four infants (3 males, 1 female) were followed weekly from the age of 3 weeks up to the time they acquired 5 weeks of reaching experience. The method and data in this report focus only on the touch activity that occurred while infants were in supine during the pre-reaching period, that is the period spanning from 3-weeks-old until infants acquired head control (between 9 and 13 weeks of age). Potential participants were referred to us via an OB/GYN practice at the University of Tennessee Medical Center in Knoxville, TN, United States before the infants were born. The principal investigator (DC) met with the expecting parents to explain the goal of the study and methods used. If parents agreed to participate in the study with their infant, they signed a consent form and began to come to our Infant Perception-Action laboratory 3 weeks after their infant was born. One infant (♀) started the study at 4 weeks old and that same infant dropped from the study when she was nearing head control. Her parents were no longer able to bring her to the weekly sessions. Thus, this infant only provided touch data during the pre-reaching period. Also, all infants had one missing data collection session at some point in the study due to sickness. All four infants were born full-term, two via C-section. They weighed between 2693 and 3629 g at birth. Three of the four infants had APGAR scores of 8 and 9 at 1 and 5 min, respectively, after birth, one infant (♂) had APGAR scores of 3 and 5 at 1 and 5 min after birth but showed no neurological disorders or developmental complications during his follow up. Three infants were White, one was of Hispanic descent. Parents received a $25 gift card at each visit and on their last visit, they also received a copy of all video records and a baby book of pictures of their child taken while in the laboratory. This study was approved by the Institutional Review Boards of the University of Tennessee and Medical Center.

### Materials

**FIGURE 1:**
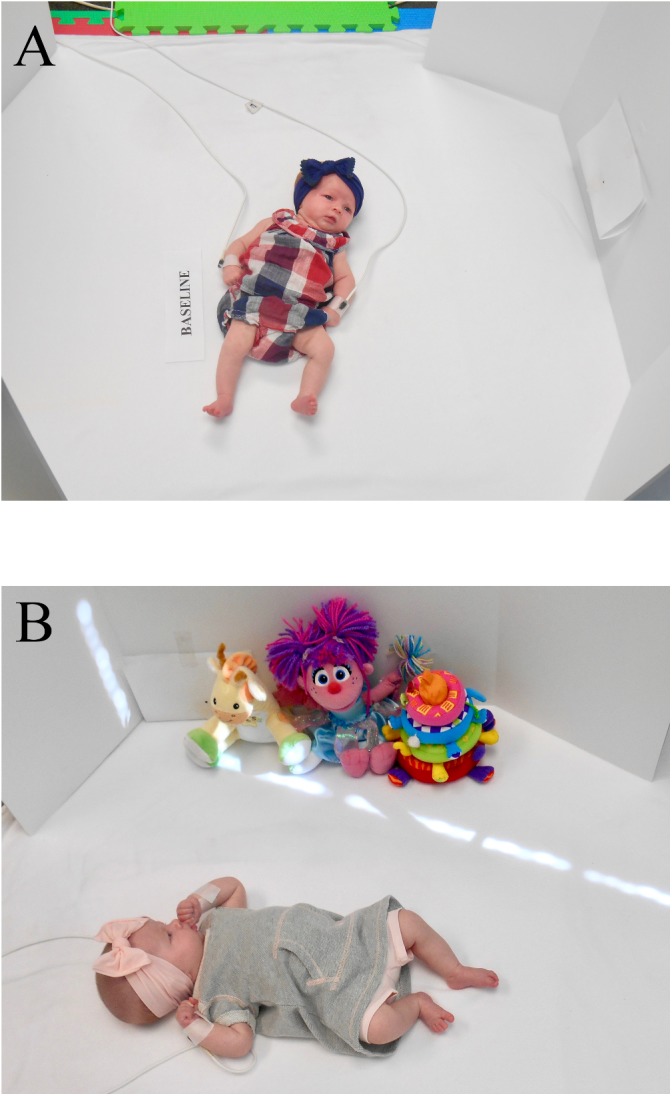
Recording setup. **(A)** Baseline condition, **(B)** toys-in-view condition. Written informed consent was obtained from the legal guardian of the infant for the publication of these images.

An all-white foam, uniformly flat and padded surface measuring 126 cm × 129 cm was placed on the laboratory floor to support the infants during the recordings. Two vertical white panels (91.5 cm × 122.5 cm) standing on each side of the infants were used to block distractions from the surroundings (see Figure 1). Two digital videos (Panasonic PV-GS39), one recording from above and the other recording from the front were fed in a Digital Video Switcher SE-500 (Datavideo Corp., Whittier, CA, United States) providing a split-screen image of the two video images. These video recordings were captured on a Dell Optiplex 9020 via an Osprey 820e digital video capture card (ViewCast Corp., Plano, TX, United States) and recorded with the Debut Video Capture software (NCH Software Pty Ltd., Australia). Both split images provided a simultaneous full view of the infant body.

Objects used for the toys-in-view conditions were a fairy doll, a giraffe, and a ring stacker all made of soft cloth and colorful material (see Figure 1B). These objects measured between 19 and 23 cm in height and between 10 and 24 cm in width. The giraffe could play infant lullabies, but only when pressed on the tummy. A set of Dr. Seuss books and an infant mobile were also used for some of the testing conditions, but those conditions will not be reported in this manuscript.

In addition, the infants were wearing 8 mm markers attached to the dorsal side of their wrists with hypoallergenic Johnson and Johnson soft cloth tape. The markers were part of an electromagnetic motion analysis system (Flock of Bird, Ascension Technology Corp., Burlington, VT, United States) that was used to record the infants’ arm movements. However, because the analyses reported in this manuscript focus mainly on touch activities, no movement kinematics analyses are included in this report.

### Procedure

The data collection sessions were scheduled at regular times during the weeks that were convenient for the parents and corresponded to wake times for the infants. Infants were brought to the laboratory following feeding times to ensure that they were alert during testing. Parents were not instructed to alter the clothing of the infant, and the observation proceeded with the clothing on that the infant wore into the lab. The clothing varied depending on the season ranging from onesies, dresses, and long sleeves with long pants. After birth, infants typically wear clothing throughout most of the day, thus leaving the clothing on the infants during our observations provided a naturalistic context closer to how infants normally experience their body on a daily basis. The current report focuses on the development of touch patterns in a baseline condition and a condition with objects in view. Recordings always began with the baseline conditions first during which the infants were placed in supine in the middle of the padded surface. No stimuli were presented during this condition (see Figure 1A). The toys-in-view condition immediately succeeded the baseline condition by placing the three objects (doll, giraffe, and ring stacker) parallel to the infants, at an out of reach distance of 43 cm (to not obstruct infants’ hand paths), on the side infants displayed preferred head turn (see Figure 1B). The side of the infants preferred head turn was determined during the baseline condition. During recording, infants were free to move their arms and legs at their will. If they started to show signs of fussiness, parents were allowed to give them a pacifier, although in general, the use of pacifier was avoided as much as possible. Giving or adjusting the pacifier were the only instances where parents were allowed to intervene during the recordings. Each condition lasted 5 min, except for 1 week for one infant, and 1 week for two other infants where recordings were shortened to 3 and 4 min, respectively, in response to infant fussiness.

Three additional conditions were collected (a musical condition with the objects on the non-preferred head turn, a parent reading condition, and an overhead mobile condition). Touch in these conditions has not been analyzed yet. They were introduced mainly for the purpose of measuring changes in overall movement activity as a function to parental/musical sound, which is not the focus of this paper. On two out of the 32 weeks video recorded, infants received the mobile condition after the baseline, instead of the toys-in-view condition. This switch in condition was done in response to infant fussiness.

### Touch Coding and Analyses

**FIGURE 2:**
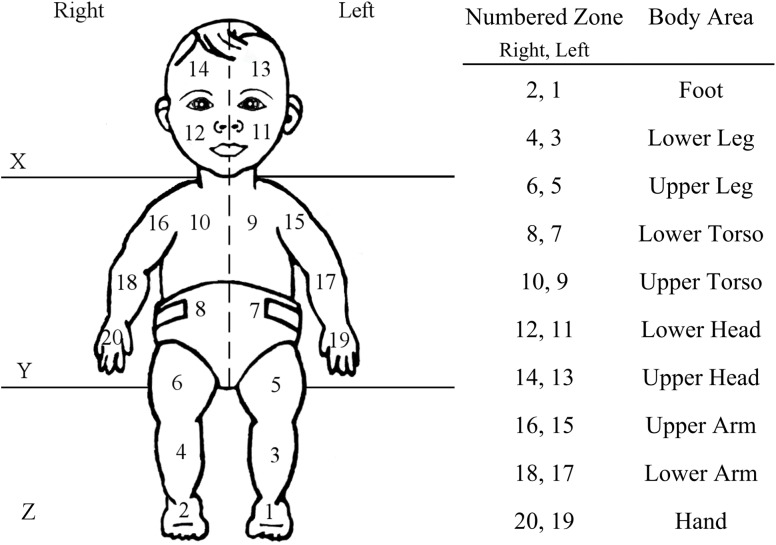
Map of body and floor areas used for the coding of the touch locations. The body was divided into 20 areas corresponding to specific body parts. The floor was divided into 3 broad areas (*X*, *Y*, *Z*) respective to the head, trunk, and legs of the infants. Body and floor were divided vertically into a right and a left side.

**FIGURE 3:**
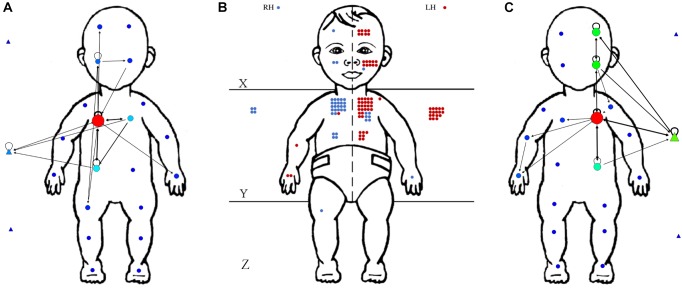
Illustration of number of touches coded by area and body side with corresponding network maps from one condition and session (week 6) of one infant (DJ). **(A)** Network map of touches and transitions performed by the right hand. The colored dots (nodes) represent the different locations touched, their size and color indicate the frequency each were touched (warmer colors indicate more frequent touches to that area), and the arrows and their thickness indicate the direction and frequency of transitions between pairs of nodes. **(B)** Frequency of touches by area. Each dot corresponds to a coded contact to that area. The blue dots are contacts performed by the right hand, the red dots are contacts performed by the left hand. **(C)** Corresponding network map for the left hand.

The coding of the videos was performed with the data video coding software Datavyu v1.2 (Datavyu Team, Databrary Project, New York University). The videos were scored continuously for the onsets and offsets of touches on the body and on the floor, respectively. Self-touches to the body were identified according to a body map of 20 areas (see Figure 2). The floor (or supporting surface on which the baby laid) was sectioned into three additional areas (see Figures 2, 3B). Each hand was coded in separate passes. From the onset/offset of touches, we derived the duration of the touches (in milliseconds), as well as the duration when the hands were not touching the body or floor, we identified the area(s) of the body or floor where the touches occurred, and their frequency. For this coding, if a touch occurred in a continuous manner over more than one area of the body or the floor (for example, if the hand moved from head to trunk while maintaining contact with the body), it was counted as a single “complex” touch, but the different body/floor areas covered during such more complex touches were recorded. Likewise, depending on the analyses, the duration of those more complex touches was either considered as a single continuous touch with one duration, or the touch duration was split evenly across areas touched. Touches were not considered if they were shorter than 280 ms (7 video frames), or if they occasionally occurred in contact with the parents’ hand (for example when the parent adjusted or gave the pacifier to their infant). Infants’ hand contact with the parent hand occurred rarely. A code of unknown was also used for times when the infant hand could not be seen, and it was impossible to determine if a touch occurred. Unknown codes only represented 2.2% of the total video footage recorded across the 4 infants. Finally, touches to the mouth (as opposed to touches to other areas of the head) and touches on bare skin (as opposed to touches on clothing) were coded in separate passes.

The touch coding was performed by 3 trained coders who worked independently. They each coded a 3rd of the entire video footage while ensuring that 20% of the videos were coded independently by all three pairs of coders to assess reliability coding among them. The weeks and infants were assigned randomly among coders. Interrater reliability scores for onset/offset of touches (with a 7-frame margin of error) were 80.3% for the left hand, 79.42% for the right hand (*r* = 0.980). Interrater reliabilities for the areas touched were 83.62% for the left hand and 85% for the right hand (*r* = 0.875). Touches to the mouth corresponded to 98.77% interrater agreements and touches to the skin yielded a 93.16% agreement.

We used the Social Network Analysis and Visualization software SocNetV v2.4 (Dimitris V. Kalamaras©, 2005–2018) to quantify the number of transitions (or connections) between body areas and supporting surface locations touched (nodes), to determine the centrality node (the body area from which most touches left), and to measure the network density, which captures the portion of potential connections in a network that correspond to actual connection (see Figure 3). As the number of connections across nodes increases, so does the network density. For this analysis, each area covered by complex touches was represented on the network map.

Our data met normality distribution assumptions. However, given the few missing weeks and the fact that not all infants were followed for the same duration, we used Linear Mixed Model (LMM) ANOVAs with a Bonferroni adjustment for multiple contrasts to analyze the trends in the data. All 4 infants provided data up to weeks 9 of age, one infant provided data up to week 12 and one infant was followed until week 13 of age. Weeks 10 and 13 ended up being excluded from our analyses because those weeks only had data for one infant. However, for the purpose of visualizing the data, these weeks are represented in our graphs. The symbols and lines correspond to those specific weeks excluded from the statistical analyses appear in gray on our graphs.

## Results

### Durations of Hands on Body, Floor, or in the Air

**FIGURE 4:**
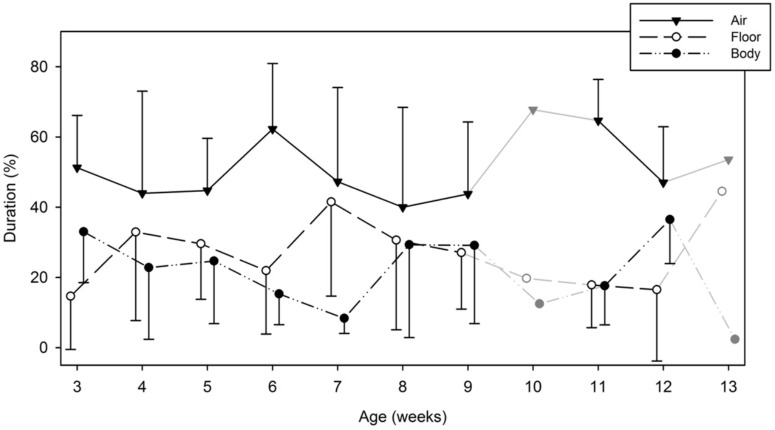
Average and standard deviation of the proportion of time that infants spent contacting the body, the floor, or moving their hand in the air from one area to the next by week. On weeks 10 and 13, the symbols and lines are grayed to indicate that only one infant contributed data on those specific weeks. Data from these specific weeks were not entered in our statistical analyses.

Our first analysis was to assess where infants kept their hands the longest: in contact with their body, in contact with the supporting surface (floor), or in the air while transitioning from place to place. Figure 4 shows the average percent of time all four infants spent in each of these broad locations by week. A Condition (2) × Hand (2) × Location (3) × Week (9) Linear Mixed-Model ANOVA revealed a main effect of location [*F*(2,228) = 45.249, *p* < 0.0001], but no main effects of condition, hand, or week. Infants spent significantly more time with their hands in the air moving it from one location to another, than either touching their bodies or the floor. This was true during both the baseline and toys-in-view conditions, and occurred similarly with either hand. An interaction between weeks and touched areas was also significant, [*F*(16,228) = 2.77, *p* = 0.001]. Pairwise comparisons indicated that in the earlier and later weeks, infants spent relatively more time with their hands in the air compared to touching their body or the floor, however, in the middle period those differences were much smaller (*p* < 0.0001). Of the touches that infants made to their body, 45.11% were on bare skin locations, while the remaining 54.89% were on parts of the body covered with clothing.

### Network Density, Number of Nodes Touched, and Point of Centrality

In order to understand the complexity of how the infants distributed touches to their body and the floor, we created a network map to analyze the areas contacted by each hand, their densities and transitions. Figure 3 provides an example of a network of touches that were exhibited independently by the right (Figure 3A) and left hand (Figure 3C) in one infant during the same week and condition (infant DJ, week 6, baseline condition). Figure 3B represents a map of frequency of touches as distributed across the 20 areas of the body and three areas of the floor. On this frequency map, each dot represents a touch to an area and the color indicates if the touch occurred with the left hand (red) of right hand (blue). Transitions between these touched areas were obtained from the temporal sequence of touches coded through Datavyu and subsequently entered in the SocNetV program to create a network map. On Figures 3A,C, each dot (on the body) or triangle (on the floor) are “nodes” and represent areas where contacts occurred. The size and color of the nodes reflect how often those areas were touched: larger and “warmer” colored nodes reflect more touches to those areas. The arrows, and their directionality and thickness, represent the transitions from one node to the next. These are called “transitional arcs.” Thicker lines correspond to more frequent transitions between nodes. Measures of network density and transitional arcs by week and by hand were obtained by dividing the number of observed connections in the network by the total number of possible connections between nodes. Network densities and transitional arcs express similar trends using different scales.

**FIGURE 5:**
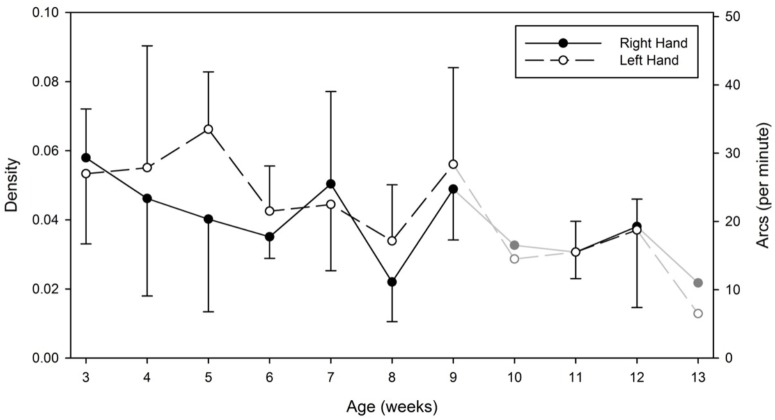
Average and standard deviation of the network densities and transitional arcs (per minute) by hand and by week. On weeks 10 and 13, the symbols and lines are grayed to indicate that only one infant contributed data on those specific weeks. Data from these specific weeks were not entered in our statistical analyses.

**FIGURE 6:**
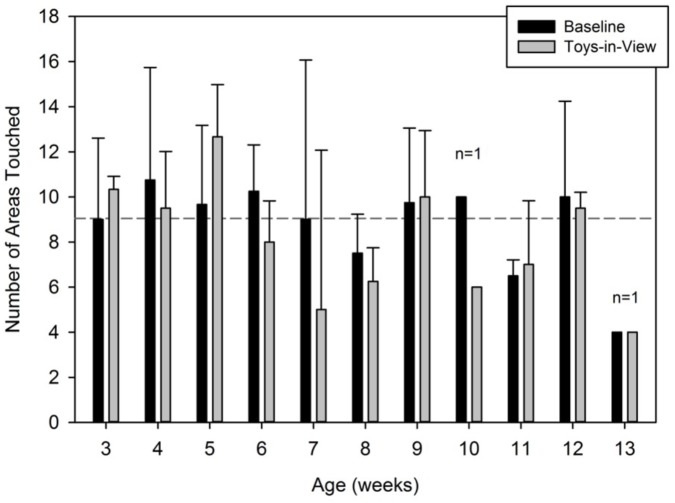
Average number of areas (body and floor) touched at least once by condition and by week. These averages correspond to areas touched by one hand. Body areas touched by both hands would double these numbers. The horizontal dashed line corresponds to the grand average across weeks and conditions.

Figure 5 shows the averaged network density and transitional arcs (per minute) across all four infants by week and by hand. The Linear Mixed Model [condition (2) × hand (2) × week (9)] revealed no significant main effects of density (and transitional arcs) across conditions [*F*(1,76) = 0.265, *p* = 0.608], and hands [*F*(1,76) = 0.957, *p* = 0.331], however, it revealed a main effect of week [*F*(8,76) = 2.627, *p* = 0.014]. Figure 5 shows that density (or transitional arcs) declined as weeks passed indicating that infants’ range of transitions across nodes lessened. On average, infants’ transitions between nodes declined from 27.25 transitions per minute (*SD* = 9.016, range = 13–42) on week 3 to an average of 19 transition per minute (*SD* = 7.89, range = 10–35) by week 12. This decline in transition number, however, did not significantly affect the number of nodes visited in the network over time [*F*(8,38) = 1.521, *p* = 0.183]. Figure 6 shows that infants transitioned on average across 9 nodes (or body/floor areas) by hand (*M* = 9.05, *SD* = 3.16, range = 6.75–11.17) in both conditions from week 3 up to they acquired head control. Statistical analyses on this measure reported no significant effects of condition or hand. Thus, the number of nodes visited over time did not change, but the routes that each hand took to transition to those nodes did.

Another measure that can capture variations in the network is the point of centrality. The point of centrality corresponds to the point on the network map where the greatest frequency of movements came to and departed from (that would be the “warmest” and largest node in the network). For example, for week 6 of infant DJ that is displayed in Figure 3, the point of centrality is the upper torso node for both hands. The points of centrality for each infant, by week, hand, and condition are reported in Table 1. This table shows that the floor was the most frequent point of centrality for all infants on most weeks, followed by the torso, the head next, and the arm on some weeks for some infants. Specifically, the floor happened to be the point of centrality 21 times (66.67%) for MA, 19 times (83.33%) for KP, 15 times (40.56%) for LN, and 14 times (56%) for DJ when we combine both hands and conditions. The torso was the point of centrality 15 times (40.54%) for LN, 9 times (36%) for DJ, 4 times for KP (16.67%) and 5 times for MA (14.7%). MA was the only infant with the head coming as the second highest point of centrality (*N* = 7, 19.44%), compared to LN, DJ and KP who had the head as point of centrality only 5 (13.31%) 1 (4%) and 1 (4.16%) times, respectively. The arms as the point of centrality occurred only twice for LN (5.41%), once for DJ and MA (4% and 2.78%, respectively) and never for LN. An exploratory Chi Square performed on these frequencies by week, condition, and hand, revealed no effects.

**Table 1:** Point of centrality by infant, hand, week, and condition.

	DJ		LN		KP		MA	
Week	Left	Right	Left	Right	Left	Right	Left	Right
**Baseline**								
3	L-Floor	U-Torso	U-Torso	U-Torso	U-Floor	U-Floor		
4	L-Torso	L-Floor	L-Floor	L-Floor	U-Floor	U-Floor	L-Head	U-Floor
5			L-Floor	L-Floor	U-Floor	L-Floor	L-Head	U-Floor
6	U-Torso	U-Torso	L-Head	U-Floor	U-Torso	U-Floor	U-Torso	Arm
7	L-Floor	L-Floor					U-Floor	L-Floor
8	L-Floor	L-Floor	U-Torso	L-Floor	U-Torso	U-Floor	L-Head	L-Head
9	*Arm*	U-Head	U-Floor	U-Floor	L-Floor	U-Torso	U-Floor	U-Floor
10			L-Torso	L-Floor				
11			L-Torso	L-Floor			L-Floor	L-Torso
12			L-Torso	U-Torso			L-Floor	U-Floor
13								
**Toys-in-view**								
3	U-Torso	U-Torso	Arm	U-Torso	U-Floor	U-Floor		
4	L-Floor	U-Torso	U-Torso/Arm	L-Floor	U-Torso	U-Floor	L-Head	U-Floor
5			U-Head	L-Floor	U-Floor	U-Floor	L-Floor	U-Floor
6	L-Floor	U-Torso	U-Head	U-Floor	U-Floor	U-Floor	U-Torso	U-Floor
7	L-Floor	L-Floor					L-Floor	U/L-Floor
8	L-Floor	L-Floor	U-Torso	L-Floor	U-Head	U-Floor	U-Floor	L-Head
9	L-Floor/U-Torso	L-Floor	L-Head	L-Head	L-Floor	L-Floor	U-Floor	L-Head
10			L-Torso	L-Floor				
11			L-Torso	U-Torso			L-Floor	L-Torso
12			L-Torso	L-Torso			L-Floor	U-Torso
13							L-Floor	U-Floor

*U, upper; L, lower; Italic indicates contralateral point of centrality.*

### Complex Touches

We mentioned earlier that sometimes touches were not limited to one single body area. Fairly frequently, infants moved their hand while remaining in contact with their body, thus crossing more than one of our defined body/floor areas. We called these touches “complex.” We were curious to know how often these complex touches occurred in the 2-month period examined, as these touches may express a deeper and more extensive haptic exploration of the body and surrounding space.

**FIGURE 7:**
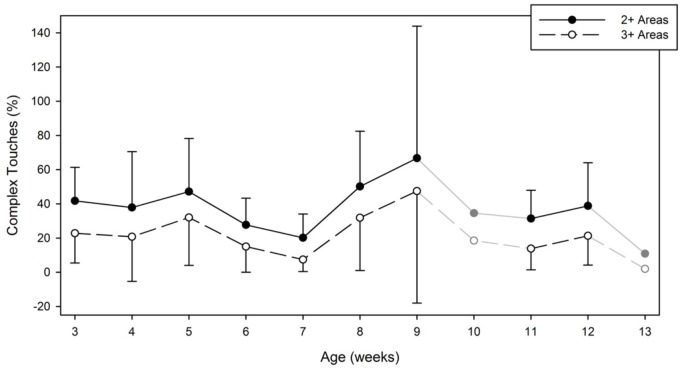
Average and standard deviation of the percentage of complex touches performed during a 5-min session by week and by number of touched areas. On weeks 10 and 13, the symbols and lines are grayed to indicate that only one infant contributed data on those specific weeks. Data from these specific weeks were not entered in our statistical analyses.

Figure 7 reveals that overall, the complex touches that contacted two or more body/floor areas represented on average 32.47% (*SD* = 18.01, range = 19.83–42.81%) of all touches. Touches to three or more areas represented 14.8% of all touches. For the two or more touched areas, a Condition (2) × Hand (2) × Week (9) Linear Mixed Model revealed a main effect of week [*F*(8,76) = 2.246, *p* = 0.033]. For the three or more touched areas, the Linear Mixed Model main effect of week remained [*F*(8,76) = 2.549, *p* = 0.016]. No other significant effects were found. The developmental trend observed was a declining one. Figure 7 shows that complex touches represented 36.6% of the touches on week 3, while they declined to 29.36% on week 12. The high percentage point observed on week 9 was due to one infant (DJ) who performed an unusually high number of complex touches on that particular week. Analyses comparing the duration of those complex touches with those of simple touches (those limited to only one specific area) revealed no differences. In other words, touches to one area were as long as touches to several areas.

### Frequency and Duration of Touches Between Body and Floor

How many touches altogether did infants perform in the 5 min observations? Table 2 reports the average number of individual contacts to the body and floor per week (collapsed across areas and hands) for all 4 infants and between conditions. This table shows that regardless of condition, on any week, infants maintained an overall high level of touches [*baseline* grand average = 113.17, *SD* = 48.90, range = 85–162.5, *M* rate (per minute) = 23.40, *SD* = 10.2, range = 17–35.71; *toys-in-view* grand average = 105.23, *SD* = 45.27, range = 66.75–158, *M* rate (per minute) = 21.72, *SD* = 9.13, range = 15.3–29.1].

**Table 2:** Average number of touches by week and by condition.

	Baseline		Toys-in-view
Week	*n*	M (*SD*)	M (*SD*)
3	3	145.33 (58.71)	145.33 (58.71)
4	4	95.00 (71.54)	76.50 (34.66)
5	3	121.67 (46.70)	129.33 (55.10)
6	4	102.50 (21.48)	77.75 (25.81)
7	2	97.50 (72.83)	107.00 (55.15)
8	4	96.50 (34.37)	66.75 (20.27)
9	4	122.75 (68.02)	124.75 (48.31)
10	1	85.00	150.00
11	2	162.50 (30.41)	158.00 (26.69)
12	2	130.00 (74.95)	89.00 (9.89)
13	1	94.00	92.00
Grand average		113.17 (48.9)	105.23 (45.27)

**FIGURE 8:**
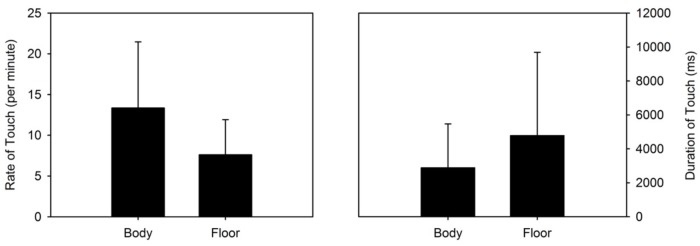
**(Left)** Mean rate of touches and standard deviation by body vs. floor location. **(Right)** Mean duration of touches and standard deviation by body vs. floor location.

When we distinguished touches between those performed on the body and those performed on the floor, we found that touches were more frequently directed to the body (rate per minute: *M* = 13.35, *SD* = 8.11, range = 8.7–18.7) than to the floor (rate per minute: *M* = 7.61, *SD* = 4.31, range = 5.13–11.85; see Figure 8, left graph). The Linear Mixed Model [condition (2) × location (2) × week (9)] revealed that location yielded a significant main effect [*F*(1,76) = 18.704, *p* < 0.0001]. No other main effect or interaction was significant.

When we examined the average duration of each touch (not the frequency of touches), we found the opposite trend. Touches to the floor, although relatively less frequent than touches to the body, were on average of longer duration (milliseconds: *M* = 4777.95, *SD* = 4910.736, range = 1731.67–8432.15) than touches to the body (milliseconds: *M* = 2880.27, *SD* = 2587.96, range = 1409.06–4079.65). Again, a Linear Mixed Model [condition (2) × location (2) × week (9)] revealed that the durations of touches between body and floor areas were significantly different [*F*(1,78) = 4.549, *p* = 0.036; see Figure 8, right graph]. No other main effect or interaction was significant. Thus, while touches to the body were more frequent, they were of lesser duration.

### Frequency and Duration of Touches Between Left and Right Hand

**FIGURE 9:**
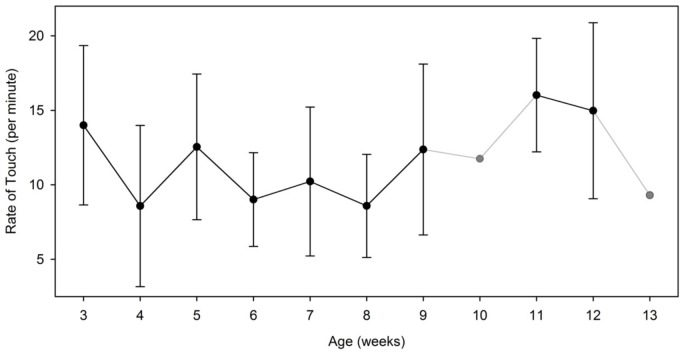
Average and standard deviation of the rate of touch (per minute) performed by week. On weeks 10 and 13, the symbols and lines are grayed to indicate that only one infant contributed data on those specific weeks. Data from these specific weeks were not entered in our statistical analyses.

When we computed the number and duration of all touches (body and floor combined) by hand and across infants, the Linear Mixed Model analyses [Condition (2) × Hand (2) × Week (9)] revealed no lateral differences by week or condition. There were no significant differences in the rates of touches performed between the left hand (*M* = 12.03, *SD* = 5.90, range = 9.03–16.65) and the right hand [*M* = 10.56, *SD* = 4.52, range = 7.42–15.40; *F*(1,76) = 1.916, *p* = 0.17]. Likewise, the durations of touches between the left (milliseconds: *M* = 3673.22, *SD* = 3852.13, range = 1483.97–6001.88) and the right hand (milliseconds: *M* = 3384.06, *SD* = 2923.75, range = 1167.02–5131.24) were not different [*F*(1,76) = 0.185, *p* = 0.669]. However, when we collapsed the rate of touch across hands, the Linear Mixed Model revealed a main effect of week [*F*(8,76) = 3.35, *p* = 0.002]. Figure 9 shows that the overall rate of touch, whether to the body or the floor, increased over time.

### Frequency and Duration of Self-Touches to the Body: Head vs. Torso

**FIGURE 10:**
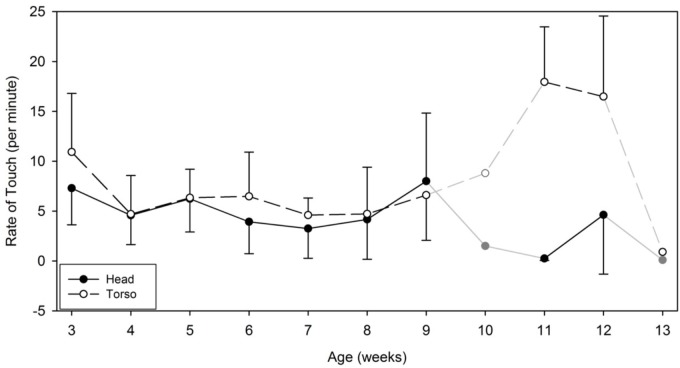
Average and standard deviation of the rate of touch (per minute) performed on the head versus the torso by week. On weeks 10 and 13, the symbols and lines are grayed to indicate that only one infant contributed data on those specific weeks. Data from these specific weeks were not entered in our statistical analyses.

Finally, our last analysis focused on the body alone and aimed at comparing differences in self-touch between head and body. The Linear Mixed Model [condition (2) × body area (2) × week (9)] performed on the rates of touch to those areas revealed that overall infants contacted their torso at a significantly higher rate (*N* per minute: *M* = 6.33, *SD* = 6.1, range = 2.8–9.48) than their head [*N* per minute: *M* = 4.10, *SD* = 4.21, range = 0.63–8.00; *F*(1,76) = 6.071, *p* = 0.015]. This body area main effect was accompanied by a significant main effect of week [*F*(8,76) = 2.62, *p* = 0.014], but no effect of condition. A significant area by week interaction further indicated that the rate of touches directed to the torso increased significantly in the last weeks of the study compared to those directed to the head [*F*(8,76) = 4.11, *p* < 0.001; see Figure 10]. This effect was driven primarily by the two infants who were followed beyond the age of 9 weeks old. Until week 9, all infants’ rate of touch to the head versus torso were not different.

In relation to the duration of touches directed at the torso versus the head, the Linear Mixed Model [condition (2) × body area (2) × week (9)] revealed no major main effects nor interactions. The durations of touches directed to the torso lasted on average 2165.94 milliseconds (*SD* = 3427.51, range = 1064.21–8532.34) and those directed to the head lasted on average 2755.60 milliseconds (*SD* = 3795.51, range = 1028.89–5370.25).

Finally, we examined how many touches to the head resulted in contacts to the mouth. The Linear Mixed Model [condition (2) × hand (2) × week (8)] performed on the proportion of touches to the mouth out of total touches to the head revealed no major effects nor interactions. In this analysis, week 11 was excluded because one of the two infants did not contact the head at all. Of the touches that occurred to the head, only 17.16% of these touches resulted in a contact to the mouth (*SD* = 0.25, range = 0.10–0.42).

## Discussion

The aim of this descriptive study was to examine the spontaneous touch activity of a few infants every week, over two 5-min time windows, from the age of 3 weeks up to the time they acquired head control – a developmental period relatively understudied. Our results revealed that from 3 weeks of age, infants actively contacted their body and the supporting surface, and they continued to do so until our observations ended. The numbers we report are in fact quite stunning. During our observations, on most weeks, infants produced nearly 200 contacts on their bodies and the supporting surface in a cumulated 10-min time period. They also spent about 50% of that time moving their arms in the air, going from one place of contact to another. This time with the hands away from any contact was significantly more than the time used to contact either the body or the supporting surface. If we multiply these numbers by the number of hours and days infants spend in a crib or playpen over a 2-month period, it becomes clear that from very early on infants receive a great deal of haptic and proprioceptive experience through their own self-generated activity.

As mentioned in the introduction, such activities are fundamental for developing an early sense of the body and for discovering the boundaries of the peripersonal space in which future developing goal-directed actions will take place. The active touches we observed were not only expressed by the high number of contacts performed, they were also indexed by the many areas that were being contacted on a weekly basis. Infants contacted roughly as many as 8–10 (out of 20) different areas on each side of their body with each hand on most weeks. The number of body areas contacted is double if we combine the number of touched areas from both hands. The untouched areas were on the 2 nodes on the bottom of the legs and the bottom floor areas, the only places that infants of those ages could not reach to. In other words, combining both hands, infants contacted all the possible body areas that were within their hand reach. Each hand mostly contacted body and floor areas that were ipsilateral to the hand making the contact, although contralateral touches occurred occasionally.

Our skewed coding scheme, which divided the floor only into three broad areas, compared to the body that was divided into a more detailed map, may give the false impression that touches to the body were more numerous than those to the floor. But the analysis comparing the overall rate of touches between floor and body independently of the area divisions confirmed that the body was touched at an higher rate than the floor. Interestingly, however, the durations of the contacts on the floor were longer than those performed on the body. The floor was also a frequent point of centrality for all four infants, followed by the torso as the second point, indicating that contacts to the body were frequently interspersed with contacts to the floor. Thus, infants explored their body with frequent touches, they explored their bodies widely by touching many body areas (mainly their head and torso which were areas within arm’s length), and they explored their body in relation to the supporting surface.

The meaning of more frequent touches to the body compared to longer touches to the floor is hard to discern given the descriptive nature of our study. But one can speculate on the range of explanations that could account for such findings. More frequent but shorter touches to the body could be more self-stimulating or self-soothing. These body-oriented touches indeed provide redundant information between the hand and body part that are being simultaneously contacted. Self-stimulation could have a significant value for young babies who initially have poor visual acuity and whose sensorimotor experiences are mainly centered around their body. Touches to the floor, on the other hand, may be more novel. These are contacts with a foreign surface never experienced before birth, whereas self-contacts with the body have clearly been experienced extensively before birth, particularly during the last months of gestation when space in the womb is tighter. Novelty with floor contacts may also entail novel arm and body postures, causing at times the stretching of muscles, compared to the more familiar limb flexions. Thus, stretching may cause new body sensations that were seldom experienced in the last months of gestation. Less frequent but longer touches to the floor may also express more relaxed states, periods of rest in between periods of active body exploration. Clearly more studies will be needed to better understand the nature of these differences in touch between surface and body.

We observed few developmental changes in the above measures, indicating that the frequent touches and active motion of the arms from place to place were an ongoing constant in those infants during their 2 first months of life. Thomas et al. (2015) who followed infants from birth to 24 weeks of age, also did not report many developmental changes during the early period. Most changes in self-touch that they observed seemed to start occurring between 12 and 14 weeks of age. In our study, however, where we documented every single touch over much longer 5-min recording periods than Thomas et al. (2015) did, we found an increase in total number of touches from 3 to 12 weeks of age. During that time period, the network density decreased. In fact, this increase in the number of touches and decrease in network density over time appeared to be related to a particular type of touches that we categorized as complex touches. We defined complex touches as those touches that transitioned over more than one body area while the hand remained in contact with the body/floor. We found that infants produced proportionately more of those complex touches in the early weeks than the later ones, which accounted for the higher network densities and lower touch count observed in the early weeks. Indeed, according to our coding scheme, complex touches counted for one touch when they occurred, but they translated into more than one area of contact when we tracked the spatial areas where contact occurred. The fact that infants produced proportionately more of those complex touches during the earlier weeks of life could possibly reflect a different kind of body exploration where the arm movements and haptic feedback being received simultaneously offer redundancy or an enhanced sensory experience that could well contribute to initially defining the body, its different parts, and their position in relation to the supporting surface. It is also possible that the greater flexor activity of young infants in their first weeks of life is at the origins of this greater number of complex touches observed early in life (Gesell, 1946). As infants progressively learn to extend their arms away from their body, complex touches, in turn, are expected to decline in number.

In line with Thomas et al. (2015), we saw a developmental change in the rate of touches to the torso and the head areas. Initially, the infants touched the torso and head as frequently, but the two infants who were followed beyond 9 weeks of age displayed a significant change in their distribution of touches between head and torso at weeks 11 and 12. Touches to the torso increased while touches to the head decreased. This transition coincides with the observations of Thomas et al. (2015). At around that same age range, these researchers noticed that touch became more caudal and was directed more to the lower body areas. Clearly, more observations with more babies during this age period are needed to further substantiate this transition. For now, we can only speculate as to what may have caused this transition. One possible explanation could be linked to the significant changes in the visual system and visual attention that occur during this age period [(Colombo, 2001); see also Corbetta et al. (2018) for a review]. As infants direct more visual attention to the surrounding world, they may direct their hands or relax their limbs more frequently along their torsos.

Infants touched body areas covered by clothing nearly as much as bare skin areas, thus wearing clothing did not appear to influence self-touch activity to bare skin areas. Further, the most represented point of centrality on the body was the torso, an area covered by clothing. We were also surprised by the low rate of touches to the mouth. Given the literature on hand-to-mouth behavior (Butterworth and Hopkins, 1988; Rochat et al., 1988; Rochat, 1993) and the recent demonstration that the mouth in 60-day-old infants is indeed a very sensitive area of the face (Meltzoff et al., 2018b), we expected touches to the mouth to be much higher. One possible explanation for our result is that studies on hand-to-mouth focused specifically on that particular behavior, while our observations documented all touches to all reachable body areas. When considering the rate of mouth touches within the realm of all touches preformed to the head, we found that touches to the mouth were not as frequent as one would expect. This finding, however, should be put to further scrutiny.

We found no discernable effects of condition or laterality. Infants moved their arms and touched their bodies and supporting surface as much when toys were in view as when not in view. As a group, they also displayed no evidence of lateral differences between hands in either self-touch, surface contact, or time spent with the arms moving from place to place. By 60 days old, infants already show hemispheric somatosensory responses from haptic stimulation to the contralateral hand indicating that body lateralization is already somehow represented in their brain (Meltzoff et al., 2018b). But spontaneous movements of the arms are different from receiving a local stimulation on the skin surface of the hand, and it is possible that despite contralateral somatosensory representation of the hand, infant have not yet established a selective motor dominance for hand use by 2 months of age. Studies that have examined lateral differences in hand movements during the pre-reaching period have reported no preference in arm activity, whether activity differences were assessed by movement count or kinematic recordings (Lynch et al., 2008; Jacobsohn et al., 2014). Further, even though infants in our study displayed head turn preferences, especially during the first weeks, these head turns, as a group, did not seem to have affected touches differentially between hands. However, it is possible that individual head turns may have had an impact. This is something we are planning to examine in future analyses.

The fact that toys in view did not affect touch patterns, their rate, duration, or location during the 2-month period was more surprising. Our objects were brightly colored and stood out from the uniform white background. But it is possible that during this very early period, when vision is poor and arm activities are mainly centered around the body, colored objects in the visual field may not be so relevant. Furthermore, our objects were static and as a result may have failed to capture the attention of the infants at an age range where object motion is important to trigger a behavioral response (von Hofsten, 1982).

The present study provided detailed information on the touch activity of infants while in supine during the 2 first months of life – a period that has not previously been extensively studied. It is assumed throughout the manuscript that these early touch activities directed to the body and the supporting surface play an important role in providing a sense of the body and an emerging sense of the self that are essential for the development of future interactions with the physical and social world. We found that from the age of 3 weeks, infants engaged in extensive touch activities of their bodies and the supporting surface on which they lay and continued to do so until they attained head control. This study was limited to intensive observations of 4 typically developing infants followed weekly. Future studies could expand on these initial observations by documenting this activity in non-typically developing populations and over larger samples of infants to assess the impact of these early embodied experiences on the formation of future goal-directed behaviors. Future studies could also track infants over a more extended developmental period, more postures, and varied conditions to obtain a comprehensive depiction of how touch experiences may contribute to infant development. Also, in this study, we shifted infants in a different paradigm as soon as they acquired head control to capture the emergence of reaching, but it remains to be seen if the intensity of their touch activities would have changed more readily after this 2-month transitional period where vision, head control, and attention all show important changes. We encourage researchers to examine more in-depth the behaviors of infants in the first months of life as they are foundational to future sensory, motor, and cognitive development.

## Ethics Statement

This study was carried out in accordance with the recommendations of the Institutional Review Boards of the University of Tennessee and Medical Center in Knoxville, TN, United States. All parents of the infants gave written informed consent in accordance with the Declaration of Helsinki. The protocol was approved by the Institutional Review Boards of the University of Tennessee and Medical Center in Knoxville, TN, United States.

## Author Contributions

DC lead principal investigator of this project, designed and oversaw every aspect of the study, including data collection and analysis, and was majorly involved in the writing of the manuscript. AD, JC, and MC collectively contributed to data collection, provided major contributions in refining the coding scheme for the touches, coded the touches from the videos in Datavyu, helped with many aspects of data processing, and provided first drafts on sections of the manuscript.

## Conflict of Interest Statement

The authors declare that the research was conducted in the absence of any commercial or financial relationships that could be construed as a potential conflict of interest.
